# Revisiting the four core functions (4Cs) of primary care: operational definitions and complexities

**DOI:** 10.1017/S1463423621000669

**Published:** 2021-11-10

**Authors:** Geronimo Jimenez, David Matchar, Gerald Choon Huat Koh, Shilpa Tyagi, Rianne M. J. J. van der Kleij, Niels H. Chavannes, Josip Car

**Affiliations:** 1 Centre for Population Health Sciences (CePHaS), Lee Kong, Chian School of Medicine, Nanyang Technological University, Singapore, Singapore; 2 Health Services and Systems Research (HSSR), Duke-NUS Medical School, Singapore, Singapore; 3 Saw Swee Hock School of Public Health, National University of Singapore, Singapore, Singapore; 4 Department of Public Health and Primary Care, Leiden University Medical Center, Leiden, The Netherlands

**Keywords:** basic concepts and models, continuity, coordination, core functions, health care organisation and management, primary care

## Abstract

**Background:**

The four primary care (PC) core functions (the ‘4Cs’, ie, first contact, comprehensiveness, coordination and continuity) are essential for good quality primary healthcare and their achievement leads to lower costs, less inequality and better population health. However, their broad definitions have led to variations in their assessment, in the innovations implemented to improve these functions and ultimately in their performance.

**Objectives:**

To update and operationalise the 4Cs’ definitions by using a literature review and analysis of enhancement strategies, and to identify innovations that may lead to their enhancement.

**Methods:**

Narrative, descriptive analysis of the 4Cs definitions, coming from PC international reports and organisations, to identify measurable features for each of these functions. Additionally, we performed an electronic search and analysis of enhancement strategies to improve these four Cs, to explore how the 4Cs inter-relate.

**Results:**

Specific operational elements for first contact include modality of contact, and conditions for which PC should be approached; for comprehensiveness, scope of services and spectrum of population needs; for coordination, links between PC and higher levels of care and social/community-based services, and workforce managing transitions and for continuity, type, level and context of continuity. Several innovations like enrolment, digital health technologies and new or enhanced PC provider’s roles, simultaneously influenced two or more of the 4Cs.

**Conclusion:**

Providing clear, well-defined operational elements for these 4Cs to measure their achievement and improve the way they function, and identifying the complex network of interactions among them, should contribute to the field in a way that supports efforts at practice innovation to optimise the processes and outcomes in PC.

## Introduction

There is general agreement in the context of primary healthcare that the achievement of the well-known four core primary care (PC) functions (also known as the four pillars, four tenets or simply as the 4Cs of PC (4Cs)), is associated with better quality services, lower costs, less inequality in health care and better population health (Baicker and Chandra, 2004; Starfield *et al*., 2005; Lewin *et al*., 2008; Chan, 2009).

The 4Cs of PC are defined as (Prates *et al*., 2017):

- First contact – access and use of health services whenever necessary;
- Comprehensiveness – promotion, prevention, treatment and rehabilitation appropriate to the PC context;
- Coordination – the integration of all the care the user receives and needs with the other health services;
- Continuity – a professional-subject-of-care temporal relationship, leading to the establishment of strong mutual trust.

Starfield first articulated them in her 1992 book (Starfield, 1992), she then updated this work in a 1998 revision (Starfield, 1998), and then it was restated in her Millbank Quarterly Review with Macinko and Shi in 2005 (Starfield *et al*., 2005). Starfield had also evaluated a key tool in assessment of the 4Cs, the Primary Care Assesment Tools (PCAT) in her 2001 paper in the Journal of Family Practice (Shi *et al*., 2001). The value of Starfield’s work is shown by its widespread adoption worldwide, notably in Spain and South America, and more recently in East Asia. Moreover, the 4Cs have been used for designing and planning PC systems (Macinko *et al*., 2007; World Health Organization (WHO), 2018), and for developing new ways of envisioning and measuring PC, like Bodenheimer’s 10 Building Blocks of High-Performing PC (Bodenheimer *et al*., 2014), and the Patient-Centered Medical Home (PCMH) in the USA (American Hospital Association and Committee on Research, 2010) and Canada (Kiran *et al*., 2015). As a result, they have become ‘the foundation for all future elaborations of key primary care attributes’ (Bodenheimer *et al*., 2014), reinforcing Starfield’s 4Cs relevance in today’s PC, family practice and general medicine fields.

However, the broad conceptualisation of the 4Cs makes it difficult to pinpoint which elements should be targeted for improving them, leading, for example, to wide variation in their performance: some attributes, like continuity (also termed longitudinality), have been well evaluated, while first contact and comprehensiveness, evidenced weaknesses (Prates *et al*., 2017). Relatedly, despite the general endorsement of the 4Cs, these broad conceptualisations have led to substantial variations in how to operationalise their assessment in PC practice and systems (Tirodkar *et al*., 2014). For example, in the USA, it is assumed that a practice receiving PCMH status automatically meets the 4Cs, which goes against the published evidence showing problems in many designated PCMHs with first contact (eg, low number of after office hours), continuity (eg, miscommunication with PC when patient enters hospital) and comprehensiveness (eg, lack of evaluation of PC providers’ ability to provide comprehensive care themselves and abusing referrals) (Berenson and Burton, 2016).

Given the emphasis on the 4Cs and their measurement when implementing PC models (Stange *et al*., 2010), it is important to establish clear features for each of the 4Cs and include specific characteristics that may serve as guides to support improvement. Bodenheimer 2014’s study mentioned ‘blocks’ that appear to support the achievement of the essential four PC functions, such as engaged leadership and data-driven improvements (Bodenheimer *et al*., 2014), so we expand on this work by reviewing (1) definitions of the 4Cs coming from varied pieces of work and international organisations, and (2) recent evidence of specific enhancement strategies or practice characteristics associated with improvements in performance for any of the 4Cs.

Through this exercise, our aim is to provide an update for the operationalisation of the 4Cs, incorporating new aspects that may not have been considered when these features were first described, such as the role of digital technologies. In addition, we highlight PC enhancement strategies or practice characteristics that have the capacity of impacting more than one C simultaneously and the interactions among the 4Cs, so that these are considered when designing efforts to enhance PC. Given the breadth and potential scope of this undertaking, we envision this piece of work as an initial step or starting point to foster the exchange of ideas on how to think of, improve and update the understanding of the 4Cs to ultimately strengthen PC.

Box: Useful definitions

1. 4Cs: PC core functions/pillars/tenets (i.e. first Contact, Comprehensiveness, Coordination, Continuity).
2. Conceptualisation: definition of a concept.
3. Operationalisation: development of a definition for a concept that corresponds to actions that can be implemented and measured in the real world (ie, operational elements).
4. Enhancement strategies: actions intended to improve outcomes of PC (eg, IT innovations, enhancing providers’ roles, monitoring systems). These enhancement strategies can include policy-level changes, such as empanelment or change in funding mechanisms. Whether at the practice or policy level, enhancement strategies are generally intended to improve the 4Cs.

Practice characteristics: measurable features of a PC practice that corresponds to its success in achieving one or more of the 4Cs.

## Methods

Several PC organisations’ documents, including family medicine and general practice associations, and seminal papers were searched to identify definitions used for each of the 4Cs. We were able to find definitions from documents developed by organisations such as WONCA, the U.S. Institute of Medicine (IOM), the World Health Organization (WHO), the Pan American Health Organization (PAHO), WHO European Region, as well as from the work of Starfield and other experts’ key publications (eg, work from George Freeman on continuity of care (Freeman *et al*., 2003; 2007; Freeman and Hughes, 2010)). In addition, items of the PC Assessment Tools (PCAT), a well-known, validated tool to evaluate PC (Ministerio da Saude Brasil, 2010), were included in order to establish measurable characteristics of the 4Cs. The full identified definitions along with sample PCAT items are included in Appendix A.

In parallel, recent evidence was searched for identifying PC enhancement strategies or practice characteristics that were most consistently linked to each of these 4Cs. Between March-April 2019, four searches were performed using the terms ‘primary care’ combined with ‘comprehensiveness’, ‘first contact’, ‘continuity’ and ‘coordination’, in PubMed/MEDLINE. Both terms (ie, ‘primary care’ and the corresponding ‘C’) had to be present in the title/abstract. We filtered for reviews and systematic reviews to obtain summarised information on the corresponding ‘C’, and selected articles from 2013 to get the most updated evidence. The selection entailed choosing a purposeful sample of articles explicitly linking an innovation or enhancement strategy to a ‘C’, to highlight the most commonly used strategies to enhance each of the 4Cs and build the Results and Discussion sections of this study. The full summary of the selected evidence as presented in the original reviews can be found in Appendix B.

We performed a narrative synthesis, which involved consensus and critical reviews among the authors, where we derived essential and recurrent operational elements for defining and measuring each of 4Cs, and considerations about the challenge of assessing these concepts due to the interconnectedness of their functions.

## Results

### First contact (FC)

Although FC is recurrently highlighted as crucial to a high performing PC practice (ie, one that meets more care needs more effectively), there are very few reviews clarifying its definition, measurement or its independent role in changing outcomes (eg, process, health outcomes, costs, or satisfaction). In general, FC is mentioned in studies that are broadly about what PC ‘should be’ in order to best fulfil patient needs. Its definitions reinforce the idea that PC should serve as the main entry point and interface between the population and the health system (Macinko *et al*., 2007). Similarly, it is recurrently mentioned that PC should be ‘the users’ preferential contact, the main entrance door [to the healthcare system], and the PC network communication centre’ (Paula *et al*., 2016).

FC’s definitions often intermix gatekeeping functions and access. Notably, the seminal Starfield model (as operationalised in the PCAT (Appendix A)) measures both, but stresses gatekeeping. The PCAT includes these as sub-dimensions, dividing PC characteristics relevant to FC into: (1) ‘gatekeeping’, to refer to whether there are requirements to see the PC providers first, and (2) ‘accessibility’, which relates to scheduling an appointment, working days and hours of a PC facility, and to waiting times before seeing a provider. In this sense, FC implies that in order to be the point of first contact, PC must be accessible and must be the site of first contact whether by patient choice or by mandate through gatekeeping rules. In the PCAT, these are treated as independent without accounting for the fact that a PC that is inaccessible would not fulfil the objective of FC, even if gatekeeping were mandated.

In terms of evidence, efforts to improve FC include policy changes, such as empanelment (Loewenson and Simpson, 2017). Evidence regarding the ‘gatekeeping’ dimension of FC has suggested that mandating PC as the conduit to other services increases appropriate referrals, reduces hospitalisations and decreases specialist use; notably, patients are often less satisfied when denied direct access to specialists (Bashshur *et al*., 2016; Sripa *et al*., 2019). Digital/eHealth technologies, such as patient online platforms for after-hours access (Bashshur *et al*., 2016), and several workforce-related efforts, such as increasing capacity by adding non-physicians and task shifting, are associated with improved access and wider capacity of different providers to ensure access to PC (full details in Appendix B) (Leach and Hicks, 2013; Drennan *et al*., 2014; van der Molen *et al*., 2017).

### Comprehensiveness

Comprehensiveness in PC refers to the scope of services offered and its capacity to manage the most common health conditions, at any stage of a person’s life. Although comprehensiveness is consistently mentioned as one of PC’s core functions, there are not many reviews focusing solely or specifically on its impact in PC. There is literature on ways to monitor comprehensiveness.

Comprehensiveness has been described along several dimensions. These include: (1) the scope or range of services offered and available (ie, promotion, prevention, early diagnosis, curative, rehabilitative and palliative); (2) the spectrum of population needs that can be addressed along the life course, which includes the ability of practitioners to care for patients at any stage of their lives (‘cradle to grave’) and in any care setting; (3) the adopted approach to care (e.g., including psychosocial needs in a holistic approach) and (4) the depth of services (ie, severity or complexity of illness managed) and breadth (ie, acute and chronic) of conditions managed by the PC team. Each dimension is considered in the context of the prevalence of health concerns and conditions in the population served (O’Malley and Rich, 2015).

Comprehensive care definitions imply overlaps with the other C’s features. For example, to serve as an effective point of first contact, the PC provider should have the ability to receive any health problem (except for the very unusual ones) and have the capacity to either directly deal with them or have the diagnostic skills to appropriately refer the patient to a specialist (gatekeeping/coordinating role). The capabilities engendered by comprehensiveness also relate to the ability of PC to coordinate with other providers, thus being able to serve patients over the life course (relevant to continuity) (see below).

Research has examined funding strategies required to assure high levels of comprehensiveness (Prates *et al*., 2017), including panel size to optimise quality of care (Raffoul *et al*., 2016), its measurement and monitoring (O’Malley and Rich, 2015) and the importance of the ability to care for mental health problems (Spenceley *et al*., 2015). Regarding the relationship between comprehensiveness and outcomes, research indicates that more comprehensive PC is associated with greater efficiency, better health and lower costs, mostly coming from lower hospitalisation rates (full evidence details in Appendix B) (Kringos *et al*., 2010; O’Malley and Rich, 2015).

### Coordination

Coordination is one of the most widely recognised attributes of PC, and its characteristics have been highlighted in several reviews, especially in its connection to care of patients with chronic conditions, and in relationship to the use of digital health technologies. In the current health context, characterised by specialisation and information surplus, coordination is arguably one of the most challenging aspects to tackle.

Coordination in PC is described as the act of bringing together the different elements and levels of the health system for the care of a patient, both within the PC practice setting, as well as with other providers, including secondary and tertiary care clinicians. Coordination involves evaluating care needs, identifying those not performed directly by a PC provider, discussing and choosing options for fulfilling those needs with the relevant decision makers (which can include a mix of patients, families and appropriate experts), and maintaining communication among providers. Thus, coordination involves the referral and counter-referral processes within the clinical enterprise, as well as the connection to aspects ‘outside’ the health system per se, such as community and social services.

The term ‘coordination’ is sometimes used interchangeably with the term ‘integration’, as seen in the PAHO definition (Macinko *et al*., 2007). However, some publications use integration to capture a broader concept than coordination, such as in the IOM definition, where integrated care also entails the provision of comprehensive and continuous services to provide a seamless process of care (Institute of Medicine (IOM), 1996). On the other hand, the operationalisation of coordination in the Provider-PCAT treats integration as a characteristic of coordination, separating it into two subitems: ‘integration of care’ referring to general communication across all individuals involved in care (ie, by any means), and ‘information systems’, emphasizing the importance of information technology or eHealth systems for promoting effective coordination (Appendix A) (Ministerio da Saude Brasil, 2010).

The bulk of evidence regarding coordination relates to the use of shared and standardised information systems (Huitema *et al*., 2018), its relation to financial initiatives such as bundled payments and its connection to referrals and transitions from different levels of care (Loewenson and Simpson, 2017). Failure of coordination between hospitals, PC providers and community-based services has been recognised as a cause for care that is inappropriate (applied but not needed), insufficient (needed but not applied), redundant and error prone (Le Berre *et al*., 2017). Coordination has been explored in the context of cancer care (Dossett *et al*., 2017), and benefits have been shown when coordination is performed by a designated coordinator as well as by the use of digital health technologies (Samal *et al*., 2016; Vandiver *et al*., 2018; Hartzler *et al*., 2018; Falconer *et al*., 2018). The evidence also explores the way coordination can be monitored and measured (full evidence details in Appendix B) (Annis *et al*., 2016).

### Continuity

Common themes in the literature on continuity of care include the characteristics of temporal regularity, building relationships and person centeredness. Continuity of care can be seen as comprising longitudinal and personal continuity. Longitudinal continuity as ‘care given by one practitioner over a defined time’, was usually provided by the general practitioner (GP) alone; personal continuity as ‘an ongoing therapeutic relationship between the patient and practitioner; where the nature and quality of the contacts are more important than the number’(Freeman and Hjortdahl, 1997), implies the provision of care by the same team or at the same facility. Continuity emphasises the relationship over time between a patient and the provider, the patient identifies as their principal source of PC, which can be a single GP or a practice where a PC site has multiple providers.

A variety of definitions for continuity have been developed. Notably, care that is accessible, comprehensive and coordinated may be said to have high continuity. However, to include the characteristic of ‘personal relationship’, a multi-component definition was proposed, in which the central element was ‘experienced’ continuity from the patients’ point of view, achieved by a combination of a large and complex number of aspects (informational, cross-boundary, flexible, longitudinal and relational ‘continuities’) (Freeman *et al*., 2001). Subsequent definitions separated interpersonal (building trust and respect via repeated contacts with the patients, incorporating the element of choice and practice level continuity (as opposed to individual GP level) (Freeman *et al*., 2007)) and longitudinal continuity (a sense that the relationship was long-term). Then, management continuity was incorporated in the definitions, which includes ‘the processes involved in coordinating, integrating and personalizing care to deliver a high quality service’ (Freeman and Hughes, 2010), which includes providers helping patients understand their plans and treatments. A well-rounded definition of continuity, proposed by important continuity experts, emphasises the coherence and connectedness of a series of discrete health events experienced by an individual, consistent with the patient’s medical needs and characterised by the elements of care over time and focus on the individual patient (Haggerty *et al*., 2003). An effective healthcare organisation, especially in the context of PC, needs to embody the three key dimensions of continuity: informational, management and relational continuity (Guthrie *et al*., 2008). All and all, an essential aspect of continuity is the ‘perceived’ relationship between the patient and provider, giving continuity a strong subjective component.

The evidence about continuity is mixed, not in small part due to the definitions used in the studies. For example, relational continuity is linked to cost-effective personalised care and with increased patient and provider satisfaction (Freeman *et al*., 2001; Freeman and Hjortdahl, 1997). However, enforcing continuity may limit the patient’s choice and result in delayed diagnosis (Freeman and Hjortdahl, 1997; Freeman and Hughes, 2010). Deciding the level of continuity also impacts its outcomes (ie, at single provider or practice level) (Freeman *et al*., 2003), and there is a variety of elements that act as enablers for interpersonal continuity (eg, enrolment, clinician/reception staff knowing patients, ensuring sufficient consultation duration (Freeman and Hughes, 2010)) and management continuity (eg, full use of practice IT systems, availability of clinical information (Freeman and Hughes, 2010)). A recent review confirmed an association between improved continuity and lower mortality rates, although this association varies by population groups and the mechanisms by which this may occur are not clear (Baker *et al*., 2020) (full evidence details in Appendix B).

The results described here allowed us to identify several strategies or practice characteristics that are connected to two or more of the 4Cs concurrently and are summarised in Table 1.

**Table 1.** Common PC strategies/characteristics impacting PC functions

Strategy/characteristic	4C impacted	Description of impact
Enrolment/empanelment	First contact	Supports early uptake of care, improves health outcomes, enables population health approaches (Loewenson and Simpson, 2017)
	Comprehensiveness	Leads to appropriate size panels, allowing providers to deliver more timely, comprehensive care; equips providers to address individual needs of patients (Raffoul *et al*., 2016)
	Continuity	Identifying the population for which provider is responsible enhances relational continuity (Starfield, 1998)
Referrals	First contact	Requiring referals from PC leads to more approriate received care (Bashshur *et al*., 2016)
	Coordination & Continuity	Essential for coordination, supports chronic condition care, supports continuity and information flow (Loewenson and Simpson, 2017)
Digital health/eHealth/IT innovations	First contact	Added access options, supports patient-provider communication, ease of patient contacting PC provider, ease for scheduling appointments (Bashshur *et al*., 2016)
	Coordination	Allow for easier interdisciplinary, provider-provider communication; updated and available information in digital/IT systems; collaborative decision-making (Falconer *et al*., 2018)
	Continuity	Availability for patient to see provider through addditional modes (ie, telephone/email contact, teleconsult), ease for information exchange (Freeman and Hughes, 2010)
Workforce-related efforts (new roles/enhancing existing roles)	First contact	Physician assistants, community pharmacist could provide FC;(Drennan *et al*., 2014; van der Molen *et al*., 2017) nurses/PC providers trained in (e.g.) mental health/cancer enhance ability to receive this type of issues at PC level (Leach and Hicks, 2013; Zeichner and Montero, 2016)
	Comprehensiveness	Enhancing capacity of PC providers to deal with a wider range of issues (mental health, cancer, chronic conditions) (Spenceley *et al*., 2015; Zeichner and Montero, 2016)
	Coordination	Nurses/allied professionals or community health workers as care coordinators improve cost-effectiveness (Vandiver *et al*., 2018; Loewenson and Simpson, 2017); involving pharmacists in transitional care leads to higher compliance and decreases readmissions (Hartzler *et al*., 2018)
Funding/finances	Comprehensiveness	Good performance of comprehensiveness demands constant investments in physical, material and human resources (Prates *et al*., 2017)
	Coordination	Bundled payments reduce fragmentation of care for chronic conditions (Loewenson and Simpson, 2017)
Enhanced monitoring/ measurement	Comprehensiveness	Measurement of medical equiment, common health problems, technical procedures and preventive care, etc. leads to more appropriate resourcing and support (O’Malley and Rich, 2015)
	Coordination	Improved measurement of communication with patients and among staff (cross-boundary coordination), and of follow-up of tests and labs results, referrals and alerts (follow-up coordination) (Annis *et al*., 2016)

## Discussion

While the importance and conceptualisation of the 4Cs are generally accepted, their operational definitions and measures vary widely. One key difficulty is that some researchers define concepts in ways that conflate the 4Cs, trying to capture the fact that some features are only valuable if they exist with others, that is, they are not independent (additive), but synergistic (multiplicative). In order to provide clarity and uniformity, we attempted to define terms independently based on their key features, taking into account that the capabilities of a PC system to meet needs depend (1) on combinations of the 4Cs (considering their independent and synergistic effects); and (2) on the fact that different combinations of the 4Cs improve outcomes depending on context, patient population and location. Here, we provide key operational elements for each of the 4Cs (Table 2), which can be used for enhancing and/or evaluating PC in context, accounting for the additive/synergistic relationships for improving care in that context.

**Table 2.** Primary care core functions (4Cs) and suggested operational elements

4C	Operational elements
**First contact**	• Modality – how the patient interacts or accesses primary care (i.e. face-to-face interaction, and/or be via telephone, email, online appointments, teleconsultation, etc.) • Personnel involved – who is the provider receiving or engaging with the patient: a GP, nurse, care coordinator, or another team member • Level of first contact – is it defined as the patient seeing her/his individual GP or healthcare professional? Or attending her/his usual practice or clinic? etc • Conditions or situations when it is appropriate to approach primary care as the first place of contact. For example, in an emergency, it would be wiser to go directly to the A&E (linked to comprehensiveness)
**Comprehensiveness**	• Scope of services offered • Spectrum of population needs • Adopted approach to care (ie, ‘holistic’ encompassing bio-psycho-social aspects, ‘cradle to grave’, etc.) • Depth and breadth of conditions managed by the primary care team (ie, if cancer or chronic condition, to which extent can primary care handle these), based on the prevalence of health concerns/conditions in the population served
**Coordination**	• Links between: ○ Primary and secondary/tertiary levels of care ○ Primary care and social/community healthcare settings • Referral and counter-referral processes • Workforce managing coordination and transitions of care • Technologies leveraged to improve coordination (including levels of interoperability within and across different systems), and monitoring systems
**Continuity**	• Type of continuity (eg, relational, management, informational etc.) • Level of continuity (eg, individual GP or practice level) • Context for continuity (eg, person-focused or disease-focused)

These operational elements provide tangible measurement features for evaluating the achievement of a particular ‘C’ and may be considered, along with the enhancement strategies and practice characteristics in Table 1, when designing programs or initiatives aimed at enhancing PC. These operational elements may provide guidance regarding which PC practice elements should be targeted when trying to improve or evaluate a particular ‘C’ of PC.

The goal of FC is to ensure that patients have efficient access to health services and avail themselves of that access. FC should be evaluated by considering the modality, personnel involved and level of contact as well as the situations in which PC should be approached (as opposed to approaching other levels/departments). The role assigned to FC will depend on the outcomes a health system would like to prioritise (eg, faster attention vs. better relationships between providers-patients vs. patient satisfaction, etc.). While FC, in its gatekeeping function, is broadly associated with more appropriate received services (ie, patients seeing a PC provider first receive the services they need, instead of going directly to a wrong specialist) and lower unnecessary healthcare utilisation, it is also seen as a burden for patients who want and may benefit from direct access to specialists, because it restricts freedom of choice. Restricting freedom of choice may impact patient satisfaction and potential health outcomes. But freedom of choice (ie, removing FC as a gatekeeper) carries responsibility and increased costs to the system as a whole, which ultimately impacts all citizens in the form of fees, taxes or insurance premiums. Thus, we recommend that FC should not be positioned as conflicting with choice; rather the goals of FC as a gatekeeper should be incentivised (instead of mandated) while still providing choice to the patient (eg, by paying lower insurance premiums or by removing co-payments at subsequently referred levels of care).

In the current era of valued-based care, it is important to note that successful FC does not necessarily mean seeing the same GP but can also mean seeing other team members at the same facility. Additionally, the mode of FC may change given changes in technology (i.e., via the internet, phone or teleconsultation) and depending on the condition to be treated (i.e., acute versus chronic). Digital health technologies and expanded PC roles have the potential to improve FC by allowing users to engage PC more easily, especially for its access sub-component.

Comprehensive PC refers to the availability of services and capacity of providers to address most health problems of the population they serve. Its scope is defined by the interaction frequency (how common a problem is) and the complexity of a problem that can be handled at the PC level. We recommend that monitoring and measuring the comprehensiveness aspects of a PC should emphasise the capability of the PC entity to meet the health needs of the population, including not only the ability to provide specific services for specific types of patients within the clinic, but also the competence to make assessments for referral and to interpret the outcomes of those referrals.

Coordination of care is defined as the capability of PC to connect the care of a patient across the different levels of the health system and beyond. A majority of the evidence about coordination is focused on the care for individuals with complex chronic conditions, who require a range of services across multiple providers and locations. We recommend that coordination be defined and measured functionally, that is, based on the degree to which the PC level acts as the nexus of care for this population, assuring that patients navigate the healthcare system smoothly avoiding confusion, inappropriate care and unnecessary rework. Functionally defined, coordination is separate from the means or strategies used for fulfilling the coordinating function. One example is empanelment or registered patients lists; as seen also with other Cs, empanelment can facilitate coordination, though empanelment should not be considered a requirement for optimal coordination. Another example is digital health. There is a tight connection between coordination and the utilisation of eHealth technologies, as they can play an essential role in enhancing communication between different stakeholders and levels of care, making information available, assisting in decision-making and enhancing surveillance efforts, all of which improves coordination. However, we should acknowledge the possibility that high levels of coordination can be achieved without digital health technologies.

Continuity has been conceptualised in several ways and trying to enhance continuity by one definition may inhibit continuity by another definition. Applying technical solutions to improving temporal continuity, for example, may reduce the patient’s sense of interpersonal continuity. Also, continuity can be additionally ‘enforced’ via empanelment where we would find another issue with freedom of choice, with the corresponding effects described above. Thus, we recommend that continuity be described from the perspective of patients and their carers (ie, how they experience it).

### Interactions and complexities

**Figure 1.**
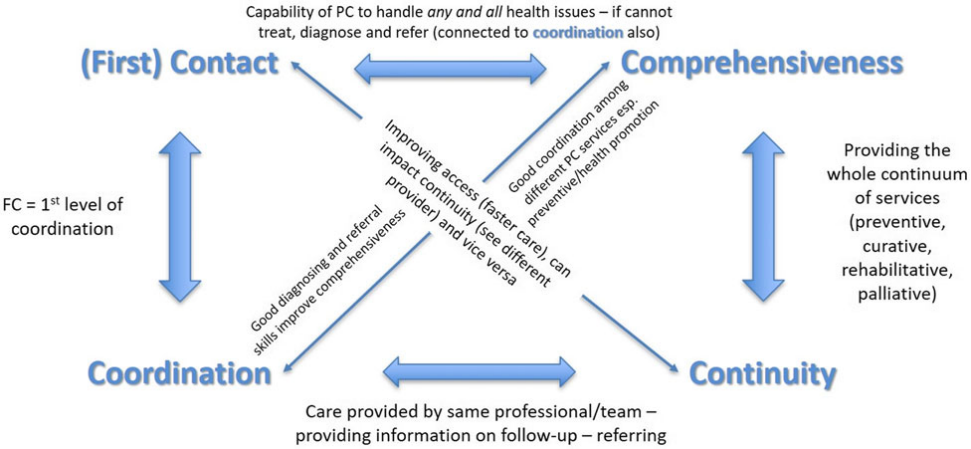
Illustration of (some of) the interrelations and complexities among the 4Cs.

A key insight from this analysis is that, to a large extent, the 4C does not operate independently and that there are several overlaps among them. In some cases, one feature can substitute for another, and in others, they may have a combined or synergistic effect. One means of enhancing one of the Cs may inhibit one or more of the others. In planning services aimed at improving the 4Cs, it is important to consider the interrelationships of these features (Figure 1).

#### FC – coordination

As the first place, the patient encounters when seeking medical services, a PC that fulfils the function of FC becomes the first point in the coordination chain. In this sense, by taking on the FC function, PC must serve as the network communication centre (Paula *et al*., 2016). Coordination (and its connection to continuity) can sometimes be at odds with the access aspect of FC. Focus on the FC function without requisite coordination may delay access and result in care that is duplicative (Quinn *et al*., 2017).

#### FC – continuity

The FC function should provide the possibility for the patient to always see the same provider(s) (same GP, nurse or team, at the same facility), and thus enhance the continuity of care from the patient’s perspective. Even when getting to see the same physician being associated with waiting for longer periods, access is reported to be positively associated with relationship continuity. Access at front desk of a GP clinic was reported to be a gateway towards good relational continuity with the patients (Freeman *et al*., 2007; Freeman and Hughes, 2010). However, when FC is implemented through a ‘gatekeeper’ mechanism, PC could be perceived as a barrier to receiving proper care, for example, in the case of a patient who wants to go directly to a specialist. In this case, having to attend to the mandated PC practitioner may be seen as a hurdle or annoyance for the patient, thus affecting the patient’s perception of her PC provider and impacting the relationship between patient and provider (i.e., relational continuity).

#### Comprehensiveness – FC/coordination

Comprehensiveness entails the capacity for receiving *any and all* health problems and providing care directly, when able and making appropriate referrals, when not. Referrals present a challenge to coordination. While the PC provider should be expected to have a core set of capabilities to provide direct service (eg, related to prevention and health promotion) (Kringos *et al*., 2010), lack of a full range of capabilities ‘in house’ can be compensated by excellent coordination.

#### Comprehensiveness – continuity

Since comprehensiveness involves the capacity to provide the whole continuum of care (ie, from preventive to curative to rehabilitative to palliative), a comprehensive PC provider would be able to assume ‘ongoing responsibility for maintaining contact with and care of the patient’ (Institute of Medicine (IOM), 1996). Thus, a PC that can provide a comprehensive range of services might more easily maintain relationship continuity (Freeman and Hughes, 2010). The challenge is that a highly comprehensive clinic may involve multiple providers, which could reduce the patient’s sense of continuity.

#### Coordination – continuity

Coordination and continuity have the potential to be tightly linked if the patient’s experience of coordination is that it is personal (i.e., the person serving as the coordinator is someone with whom the patient has a personal relationship). On the one hand, the patient may see coordination as enhancing continuity if the coordinating function is provided by someone they know and trust, or if the patient recognises that this function is being assured through the maintenance of communication in notes shared by trusted providers. Thus, when designing interventions for enhancing PC, it may be helpful to focus on assuring that the person or team members coordinating know the patient personally.

For purposes of assessing the ability of PC to meet patient needs based on the 4Cs, these interrelationships suggest that any effort to aggregate the individual features should account for the ability of high performance on some features to compensate for lower performance on others (a substitution effect), while other features must coexist at high levels to achieve the goals of each individual function (i.e., a synergistic effect).

### Limitations

This work presents several limitations, mainly related to the employed methodology. Since this was an initial attempt at redefining and updating the operationalisation of the 4Cs, we performed a superficial and quick scan of the literature and did not pursue a systematic approach for the search and selection of articles. Although this methodology still allowed for the identification of preliminary key operational features and possible enhancement strategies for the 4Cs, a more systematic and rigorous approach should result in a higher number of relevant papers, leading to more robust results and conclusions. For example, when implementing the search strategy, as described in the methods section, for articles related to comprehensiveness, the ones highlighting the importance of increasing the capacity of PC to deal with mental health issues were mostly related to dementia, underestimating the large variety of other mental health problems that PC should be able to cover, such as depression and/or anxiety, just to name a few. Searching for additional evidence via other methods was outside the scope of this study. In another example, the discussion related to gatekeeping is partially based on an interesting review, which mentions that firm conclusions could not be drawn due to difficulties in comparing studies and healthcare systems. Similarly, limiting the search to only reviews and systematic reviews from 2013 and later, done in this case to retrieve larger amounts of summarised information and relatively new enhancements, may leave out important and relevant research, which may provide valuable and additional insights.

Another key limitation relates to the scarcity of empirical evidence surrounding the 4Cs, which severely limits the ability of PC researchers and clinicians to provide evidence-based recommendations for practice and policy. For example, Baker et al.’s study (Baker *et al*., 2020) has led continuity enthusiasts to affirm that continuity of care could have ‘caused’ a decrease in mortality, when it may well be the other way around, that is, that people who choose to get better continuity may live longer for other reasons. Without clear and strong empirical evidence, coming from rigorous, well-designed studies, it would be difficult (and risky) to develop science-based guidance on how to improve PC based on the 4Cs. Recommendations for future research are provided in the following section.

### Future research

Based on the limitations described above, our recommendations for future research can be divided into two main areas. First, for further efforts at literature reviews, studies employing systematic strategies for searching and selecting articles could be developed to ensure scientific rigour and thus obtain better quality, verifiable results. Such studies could be developed separately for each of the 4Cs, delving deeper and more thoroughly on the diverging aspects related to each, which should allow for the identification of more specific operational elements and/or enhancements related to each of these Cs. Second, given the dearth of studies of the relationship between the 4Cs, health outcomes, satisfaction, and utilisation, there is a clear need for empirical evidence. Future research should focus on designing RCTs or other type of studies, to provide experimental evidence elucidating the mechanisms and causal relationships between a particular outcome (eg, lower mortality rates) and an enhanced ‘C’ (eg, improved continuity). Only this type of evidence would allow PC scientists and researchers to provide evidence-based recommendations for the improvement of PC based on influencing the 4Cs, alone or in combination, so as to produce optimal results. As not all individuals will benefit similarly from ‘enhanced’ PC, this research must account for makeup of the population served.

## Conclusion

Successful achievement of the four core functions of PC (the “4Cs”) is linked to improved population health, more appropriate use of healthcare resources and reduced costs and more generally to better-functioning health systems. In order to achieve this, it is essential to have guidance on which elements to address or measure when aiming at a particular ‘C’. The operational elements presented here will provide indications on how well the 4Cs have been achieved and how to improve the way they function. We provide clarifications for the definitions of the 4Cs in terms of specific measurable functions, separate from the means of enhancing those functions. We highlight the interrelationships between them and the importance of selecting means that tend to promote all, or at least not inhibit any, of the 4C functions. We hope that the recommendations here will contribute to the field in a way that supports efforts at practice innovation to optimise the processes and outcomes of PC.

## Supplementary material

For supplementary material accompanying this paper visit http://dx.doi.org/10.1017/S1463423621000669. click here to view supplementary material
