# Supplementary material for: Revisiting the four core functions (4Cs) of primary care: operational definitions and complexities
Source: Prim Health Care Res Dev. 2021 Nov 10;22:e68. doi: 10.1017/S1463423621000669 (PMC8581591; doi:10.1017/S1463423621000669)
Supplement: Supplementary file 1 [file S1463423621000669sup001.docx]

**Supplementary material**

**Appendix A – Primary care four core functions’ definitions and related PCAT items**

**First contact**

Definitions

*“The characteristics of the discipline of general practice/family medicine are that it […] is normally the first medical contact within the healthcare system, providing open and unlimited access to its users, dealing with all health problems regardless of the age, sex, or any other characteristic of the person concerned. […] “Normally” is used to indicate that in some circumstances, e.g. major trauma, it is not the first point of contact. However, it should be the point of first contact in most other situations. There should be no barriers to access, and family doctors should deal with all types of patient, young or old, male or female, and their health problems. General practice is the essential and the first resource.”*

The European Definition of General Practice / Family Medicine,

WONCA Europe 2011 Edition (Allen et al., 2011)

*“First contact. In a well-developed and functioning system, primary care is the usual and preferred route for entry into the health care system (although not necessarily in all circumstances). In the simplest model, the primary care clinician receives patients regardless of the disease or organ system involved and addresses a given patient's problem. […] First contact with a primary care clinician may lead to referrals to other resources—for example, to a nurse practitioner for diabetic counseling or to a cardiologist for subspecialty care. […] A derivative term is gatekeeper. In many circles, the term gatekeeper has been used to describe the function of using the experience and judgment of the primary care clinician to determine whether diagnostic tests are necessary, whether a patient's problem can be handled by the primary care practice, or whether a person needs to be evaluated or treated by another specialist or subspecialist. The primary care clinician's important role in helping the patient to obtain appropriate care in a complex health system requires a high level of skill and judgment. This judgment involves both clinical and economic decision-making.”*

Primary Care: America’s Health in a New Era,

IOM, National Academies Press 1996 (Institute of Medicine (IOM), 1996)

*“In their capacity as gatekeepers, primary care clinicians perform two broadly defined clinical roles. They serve as the entry point for patients presenting to the medical system with new health problems (i.e., first-contact care). As one of the essential attributes of primary care, first-contact care has been a traditional role for primary' care clinicians. Once health problems are identified, gatekeepers manage patients’ care by matching health needs with appropriate health care resources.”*

The Effect of First-Contact Care with Primary Care Clinicians on Ambulatory Health Care Expenditures,

Forrest and Starfield 1996 (Forrest and Starfield, 1996)

*“First contact care means that primary care should serve as the main entry point to the health system for all new health problems and the place where the majority of them are resolved. It is through this function that primary care reinforces the foundation of the PHC–based health system, representing, in most cases, the main interface between the health and social service system and the population.”*

Renewing Primary Health Care in the Americas,

Pan American Health Organization, Washington DC 2007 (Macinko et al., 2007)

Sample PCAT items (Ministerio da Saude Brasil, 2010)

*First contact, sub-item use*

- “*When you have a new health problem/need a check-up, do you go to your usual primary care provider before attending another health service?*
- *When consulting a specialist, is there a requirement for your usual primary care provider to provide a referral?*”

*First contact, sub-item accessibility*

- “*Is your PC provider/facility open on weekends/at least some days until 8pm?*
- *“When your PC provider/facility is closed, is there a number you can call if you get sick?*”

**Comprehensiveness**

Definitions

*“Comprehensive care addresses any health problem at any given stage of a patient's life cycle […] It includes ongoing care of patients in various care settings (e.g., hospitals, nursing homes, clinicians' offices, community sites, schools, and homes). Ideally, the primary care clinician listens to the patient, makes diagnoses, manages, and screens for other health care problems. The clinician educates and communicates with the patient and others who may be involved including other specialists when appropriate. He or she assumes ongoing responsibility for maintaining contact with and care of the patient and assuring that the care provided is suitable. […] Primary care clinicians receive all problems that people bring—unrestricted by problem or organ system—and have the appropriate training to manage a large majority of those problems, involve other health professionals for further evaluation or treatment when appropriate, and continue to act as advocates for their patients.”*

Primary Care: America’s Health in a New Era,

IOM, National Academies Press 1996 (Institute of Medicine (IOM), 1996)

*“Comprehensive […] care means that the range of services available must be sufficient to provide for population health needs, including the provision of promotion, prevention, early diagnosis, curative, rehabilitative, palliative care, and support for self–management. Comprehensiveness is a function of the entire health system and includes prevention, primary, secondary, tertiary, and palliative care.”*

Renewing Primary Health Care in the Americas,

Pan American Health Organization, Washington DC 2007 (Macinko et al., 2007)

*“Comprehensiveness means that all problems in the population should be cared for in primary care (with short-term referral as needed), except those that are too unusual (generally a frequency of less than one or two per thousand in the population served) for the primary care practitioner or team to treat competently.”*

Contribution of Primary Care to Health Systems and Health

Starfield et al, 2005 (Starfield et al., 2005)

*“The characteristics of the discipline of general practice/family medicine are that it […] manages simultaneously both acute and chronic health problems of individual patients; manages illness which presents in an undifferentiated way at an early stage in its development, which may require urgent intervention; promotes health and wellbeing both by appropriate and effective intervention; deals with health problems in their physical, psychological, social, cultural and existential dimensions.”*

The European Definition of General Practice / Family Medicine,

WONCA Europe 2011 Edition (Allen et al., 2011)

Sample PCAT items (Ministerio da Saude Brasil, 2010)

- “*Does your [PC provider/health service] provide: answers about healthy diet | immunizations | counselling for drugs or alcohol | sutures for injuries that need stiches | mental health counselling?*” (Patient PCAT version)
- *“Do you provide: services for all ages | counselling for making the home safe (i.e. storing medications safely) or about use of safety belts? | (for adult patients) counselling on how to deal with family conflicts | information about physical activity | how to prevent burns or falls | care for common women’s problems? | (for children) orientation on how to deal with behavioural issues | information about changes in the development of child?*” (Provider PCAT version)

**Coordination**

Definitions

*“The characteristics of the discipline of general practice/family medicine are that it […] makes efficient use of health care resources through co-ordinating care, working with other professionals in the primary care setting, and by managing the interface with other specialities taking an advocacy role for the patient when needed. […] This coordinating role is a key feature of the cost effectiveness of good quality primary care ensuring that patients see the most appropriate health care professional for their particular problem. The synthesis of the different care providers, the appropriate distribution of information, and the arrangements for ordering treatments rely on the existence of a coordinating unit. General practice can fill this pivotal role if the structural conditions allow it. Developing team work around the patient with all health professionals will benefit the quality of care.”*

The European Definition of General Practice / Family Medicine,

WONCA Europe 2011 Edition (Allen et al., 2011)

*“Coordination ensures the provision of a combination of health services and information that meets a patient's needs and specifically means the connection within and across those services and settings—putting them in the right order and appropriately using resources of the community. The goal is to focus on interactions with patient and family and their health concerns, clarify clinical care decisions, advise hospitalized patients and their families, and help patients and their families cope with the social and emotional implications of disease or illness. […] The primary care clinician also coordinates a patient's transition between health care settings—for example, hospital and home, home and nursing home, or between clinicians' offices.”*

Primary Care: America’s Health in a New Era,

IOM, National Academies Press 1996 (Institute of Medicine (IOM), 1996)

*“Integrated care is a complement to comprehensiveness in that it requires coordination among all parts of the health system, to ensure that health needs are met and that health care proceeds across time and across different levels and places of care without interruption. For individuals, integrated care involves a life–course approach to referrals and counter-referrals through all levels of the health system, and at times to other social services.”*

Renewing Primary Health Care in the Americas,

Pan American Health Organization, Washington DC 2007 (Macinko et al., 2007)

Sample PCAT items (Ministerio da Saude Brasil, 2010)

- “*Did your doctor/health service know that you have consulted a specialist? Did your doctor/health service help setup these appointments/know what the results were?*” (Patient PCAT version)

[*Coordination, sub-item integration of care*]

- “*When you refer your patient, do you provide them with written information to bring to the specialist? After your patient saw a specialist, do you talk to your patient about the results from this consultation?*” (Provider PCAT version)

[*Coordination, sub-item information systems*]

- “*Do you ask your patients to bring their medical records obtained in the past? Are patient’s records available when you are attending them?”* (Provider PCAT version)

**Continuity**

Definitions

*“Continuous or ongoing care is the longitudinal use of a regular source of care over time, regardless of the presence or absence of disease or injury. […] continuous care over time would enable the provider and patient to develop a long-term relationship.”*

Primary care: Balancing health needs, services and technology,

Starfield, 1998 (Starfield, 1998)

*“If we can agree that continuity of care without more precise definition is too broad a term to be helpful, we should limit our focus to consideration of, interpersonal continuity […] because general practice is focussed more on the person rather than on the illness. Interpersonal continuity built on repeated (but not necessarily exclusive) contacts is important in building trust and respect [and] the central skill fostered by interpersonal continuity over time is the ability to make and value a multidimensional diagnosis, based on the biopsychosocial model within the patient's context”*

Continuity of care: an essential element of modern general practice?

Freeman et al, 2003 (Freeman et al., 2003)

“*Continuity is the degree to which a series of discrete healthcare events is experienced as*

*coherent and connected and consistent with the patient’s medical needs and personal context […], and is distinguished from other attributes of care by two core elements – care over time and the focus on individual patients*”

Continuity of care: a multidisciplinary review

Haggerty et al, 2003 (Haggerty et al., 2003)

Samples PCAT items (Ministerio da Saude Brasil, 2010)

- “*When you go to your [PC facility/doctor’s office], do you see the same doctor or nurse every time? Does your doctor/nurse know you more as a person than as someone with an illness? Does your doctor/nurse know your entire medical history?*” (Patient PCAT version)
- “*In your PC facility, are patients always seen by the same doctor/nurse? Do you think your patients feel comfortable to talk to you about their concerns and problems? Do you know what medications is your patient taking?*” (Provider PCAT version)(Ministerio da Saude Brasil, 2010)

**Appendix B – Summary of selected evidence from recent publications**

**First contact**

Policies:

- Enrolment is identified as a key measure for first access, with flexibility to choose a PC practice and provision for portability. Enrolment is reported to support early uptake of care, to improve health outcomes, and to enable population health approaches (Loewenson and Simpson, 2017).
- Ensuring that PC services register their patients and populations served through enrolment is further accompanied by public education and active support for people’s uptake of services, particularly in disadvantaged communities (Loewenson and Simpson, 2017).

Referrals:

- One important aspect of the FC function relates to the concern that patients’ self-referring to specialists would lead to inappropriate referrals as well as increased cost, and the PC provider would thus limit access to specialty care. This might be controversial, though, as patients may see this as a referral barrier (notwithstanding efficient use of resources and cost containment in managed care) and perceive primary care physicians as unable to adequately care for their concerns, which would undermine patients’ trust and confidence in their primary care physicians (Bashshur et al., 2016).

Digital health innovations:

- Substantial advances in information and communication technology and related fields would enable doctors and patients to interact with each other and among themselves in ever more efficient and effective devices and platforms, not now feasible, enabling patients to contact the primary care provider more easily and effectively (Bashshur et al., 2016).
- Patients were in favor of having access to their PCP over the Internet or telephone during off-hours, as well as when seeking advice on health problems, prescription renewals, and making appointments. Some were willing to pay modest amounts out-of-pocket for such services (Bashshur et al., 2016).
- Use of the Internet had generally positive effects in several areas, including electronic scheduling appointments (29% improvement in specialty care) (Bashshur et al., 2016).

Workforce:

Additional roles in primary care and expanding capabilities of existing roles may enhance FC:

- Physician Assistants in primary care can provide triage or deal with patients with minor, self-limiting illnesses in general practices and other first-contact settings such as walk-in centres. This would help the area of patient pressure on practices and could free up the doctors’ time to deal with patients’ more complex problems or with other types of activities such as commissioning (Drennan et al., 2014).
  - However, others have argued that using the GP as the FC, particularly in triage systems, was more effective and cost-efficient owing to their expertise levels and ability to take risk (Drennan et al., 2014).
  - In some settings, such as rural areas with shortages of doctors, physician assistants become the first contact providers of primary care (Halter et al., 2013).
- Nurses could be equipped to deal with early stages of dementia, given their ideal position (patient-social environment interface) to notice the early signs and symptoms of dementia (Leach and Hicks, 2013).
- The community pharmacist can screen for COPD via completion of a validated disease risk assessment questionnaire (e.g. COPD Population Screener) and spirometry testing, based on a patient's needs when presenting to a pharmacist (e.g. advice for breathlessness) or medication needs (e.g. a cough remedy or nicotine replacement therapy). If appropriate, the pharmacist may offer lifestyle advice and refer that patient to their physician for confirmatory diagnosis and treatment (van der Molen et al., 2017).
- Enhancing PC physicians (or other roles, such as nurse practitioners, physician assistants, etc.) capability to accurately diagnose common cancers and to recognize unusual presentations of highly curable cancers (Zeichner and Montero, 2016), could enrich the FC encounter, as early detection and treatment of some cancers have high prospects of recovery/remission.

Outcomes:

- Association between gatekeeping and better quality of care, especially in terms of preventive care, and appropriate referral for specialty care and investigation. However, unfavorable outcomes reported for patients with cancer under gatekeeping, and concerns about the ability of GP gatekeepers to provide a correct diagnosis (Sripa et al., 2019).
- Gatekeeping resulted in fewer hospitalisations and lower specialist use, but in more primary care visits. Whereas some studies show that gatekeeping has lower healthcare use and expenditure, others suggest that gatekeeping has higher ambulatory care and outpatient expenditure (Sripa et al., 2019).
- Primary care clinicians have conflicting views on gatekeeping, whereas patients are often less satisfied with gatekeeping schemes, preferring direct access to specialists (Sripa et al., 2019).

**Comprehensiveness**

Systems perspective and funding:

- A good performance of comprehensiveness demands constant investments in physical, material and human resources, which requires assigning to PC its real significance and not to be characterized as a service of low complexity and requiring low investment (Prates et al., 2017).

Empanelment:

- A physician who has an appropriately sized panel can deliver more timely and comprehensive care to his or her patients, who are then more likely to be satisfied (Raffoul et al., 2016).
- Compared with PCPs who provide fragmented care (e.g., in urgent care settings), PCPs who provide continuity of care to an appropriately sized panel of established patients are better equipped to address the individual needs of their patients. They also have more time available to coordinate care with subspecialists, improve communication with their patients, provide behavior change counselling, evaluate quality, and monitor patient outcomes (Raffoul et al., 2016).

Mental health:

- PC physicians often lack knowledge about dementia, lack confidence in recognizing the symptoms, are unsure about how or if to conduct cognitive screening, face constraints in providing the time-intensive support required for patients and caregivers, lack knowledge of how to manage behavioral or psychological symptoms. […] There has been an evolution in the dementia care literature from earlier models to more recent assertions that PC needs to develop an enhanced capacity to handle more complexity in dementia care, within the context of the primary care relationship (Spenceley et al., 2015).
- Expanding the capacity of primary care to address mental health issues (e.g. dementia) is needed to improve comprehensiveness and requires:
  - services and interventions to be better coordinated around the dementia;
  - models of chronic disease management to be applied to dementia care, albeit with modifications to include the role of the caregiver in self-care because of the diminishing capacity of the patient;
  - more knowledge/education for teams involved in the primary care of people with dementia (Spenceley et al., 2015).

Measuring/Monitoring comprehensiveness:

- Primary care providers may need more resources to deliver comprehensive care, and without measurement and support for its improvement, comprehensiveness may further whither as other measured aspects of primary care improve. […] Under-measurement of comprehensiveness results from factors including the lack of a common vocabulary, patient population differences, insufficient agreement across specialties, and inadequate data sources (O'Malley and Rich, 2015).
- Features to monitor comprehensiveness should include (O'Malley and Rich, 2015):

1. Medical equipment available;
2. First contact for common health problems;
3. Treatment and follow-up of diseases;
4. Medical technical procedures and preventive care;
5. Mother/child/reproductive health care;
6. Health promotion.

Outcomes:

- A more comprehensive primary care is linked to greater efficiency, defined as a wider range of services, with better health outcomes, provided at lower costs, lower hospitalisation rates for ambulatory care-sensitive conditions, improved health and better self-reported health outcomes, and greater equity (i.e., reduced disparities in disease severity as a result of earlier detection and prevention across different populations). Without comprehensive primary care, patients experience fragmented care across numerous different specialists, resulting in higher healthcare costs, more diagnostic tests and interventions, and more types of medications (O'Malley and Rich, 2015).
- Lower hospitalisation rates for ambulatory care-sensitive conditions are associated with a comprehensive scope of primary care services. Preventive health care activities are cost-effective in the primary care setting. Early detection and prevention of progression of illness was related to reduced disparities in severity of illness. The delivery of a wide range of services by primary care providers was related to improved health. Comprehensiveness of care was positively associated with primary care strength (Kringos et al., 2010).

***Coordination***

Health system integration:

- Successful care coordination elements for health systems integration include (Huitema et al., 2018):
  - Shared and standardized information systems accessible from any point in the care network;
  - Shared care plan with clearly defined patient-centered goals of care, and mutually understood and agreed- upon provider (formal and informal) responsibilities;
  - An organisational framework clearly specifying the linkages between constituents of the care network and community-based services;
  - Clearly defined protocols to facilitate seamless transitions and navigation for patients and providers between levels and sites of care and are anchored in primary care.

Finances:

- Bundled payments have been used to enable more comprehensive and coordinated care. In the Netherlands, they were introduced in 2007 to encourage comprehensive PC, reduce fragmentation of services for chronic conditions, limit PC referrals to secondary care, and facilitate online consultations and more flexible opening hours. A single fee paid by the insurer to a contracting entity (the care group) covers the care needs of people with specific chronic conditions, such as diabetes, for a fixed period. The care group, composed of multiple health care providers, delivers or subcontracts care from various services (PC, specialists, laboratories) (Loewenson and Simpson, 2017).

Referrals:

- PC coordination of referral to secondary care and other services is argued to support the continuity of care needed to manage chronic conditions. Although this approach is negatively viewed by some as limiting choice, PC roles in referral continuity are relatively widely accepted in many high and middle income countries (Loewenson and Simpson, 2017).

Transitions through different levels of care for chronic conditions:

- Approximately 20% and 33% of patients with chronic conditions go through complicated transitions and re-hospitalisation within 30 and 90 days, respectively. Most of these unwanted outcomes can be explained by the lack of coordination and continuity during transitions. Problems with information exchange and collaboration at hospital discharge are often reported, with the PC physician receiving either incomplete, late or no discharge summaries at all. Transitional care interventions were developed to address issues in transitions of care, which encompass education on self-management, discharge planning, structured follow-up, and coordination among the different healthcare professionals, frequently involving primary care physicians (Le Berre et al., 2017).
- Transitional care reduced readmission rates and emergency department visits and improved mortality rates at 6 months after discharge. These interventions provide better continuity of care, with communication between different healthcare professionals as an included component. They also include better self-management skills, since education about chronic diseases and closer monitoring of patients through phone calls or phone availabilities was another component of most interventions (Le Berre et al., 2017).

Cancer:

- Coordination between [cancer] specialists and PCPs was listed as one of four key components of survivorship care for this population, but recommendations for how these physicians should interact during the cancer care continuum—including diagnosis, treatment, surveillance and palliation—were lacking. […] A fundamental component of the PCP-specialist relationship is communication, and the frequency, quality and ideal means of communication between PCPs and cancer specialists is poorly understood. […] PCPs need to know what to watch for, when to refer back to the cancer specialists, and that cancer specialists will respond in a timely way when patients are re-referred (Dossett et al., 2017).

Multidisciplinary teams and expanded roles:

- The utilization of multidisciplinary coordination of care was advantageous for individuals with chronic disease process, for example by using a registered nurse as the program manager and overall coordinator of care for participants (Vandiver et al., 2018).
- In many HMICs, comprehensive PC approaches are delivered by a mix of clinical, health, and allied professionals in multidisciplinary teams coordinating with public health and other services and operating also in community settings (Loewenson and Simpson, 2017).
  - For example, one of the main functions of a community health worker in PC is care coordination. This role provides information and assistance to patients about receiving care from institutions and providers outside of primary care; navigates individuals at risk for coronary heart disease by making medical referrals to local clinics and health care providers; meets with patients following each clinic appointment to help direct them to the laboratory or to other appointments (Loewenson and Simpson, 2017).
- A cost-effective workforce that includes community health worker in PC might help overburdened care teams meet the Quadruple Aim through community- based clinical services, resource connections, and health education and coaching (Hartzler et al., 2018).
- Involvement of a pharmacist was another key component of the most successful [transitional care] interventions, which might translate into improved treatment compliance, and more precise individual medication adjustment. Since a considerable number of admissions are attributable to inappropriate medical treatment, the “correction of misconceptions and lack of understanding” about medications, leading to higher compliance, can indeed be an important element in decreasing readmissions, readmission days and mortality (Hartzler et al., 2018).

Digital health innovations:

- A variety of technologies have been examined for their use in achieving interdisciplinary communication and coordination of care (Falconer et al., 2018):
  - Telemedicine aims to improve patient health through the use of real-time interactive communication between physicians and patients at distant sites using audio, video, or other electronic equipment;
  - EHRs can promote coordinated care by allowing clinicians to readily update patient health information and distribute it to other authorized providers in disparate care settings;
  - Web-based communication tools can aid in collaborative decision making and patient–provider communication by offering a means for patients to establish goals, view test results and medications, and identify care team members.
- Benefits of technology-based care coordination are evident across many areas, including screening, scheduling assessments, accessing patient information, facilitating communications, and improving treatment compliance. However, integration of technology into routine mental health care continues to remain challenging because of barriers including, but not limited to, cost, access, and usability of health (Falconer et al., 2018).
- Interoperability would allow information transfer during transitions and form a basis for many more opportunities for health IT to support care coordination. For example, when a patient sees multiple specialists these specialists need to negotiate responsibility for the patient’s medication regimen and other care. The lack of interoperability between the specialists’ EHRs may be a barrier to development of new health IT tools to support this domain of care coordination (Samal et al., 2016).

Monitoring/measuring coordination:

- Five domains related to care coordination include: ‘longitudinal continuity’, ‘relational continuity’ (defined as patient developing trust and respect for provider over time), ‘informational continuity’ (all providers have access to comprehensive patient information), ‘cross-boundary coordination’ (coordination across different health care settings or systems), and ‘follow up coordination’ (coordination related to appointments, medications, testing, or procedures recommended by initial visit) (Annis et al., 2016).
- Other measures for care coordination found in the literature can be divided into (Annis et al., 2016):
  - Care management: Pre-visit preparation; wellness promotion, disease prevention and screening, population health management; chronic disease registries, identification of high-risk patients and their needs; patient education and engagement; care plan development and progress, goal setting; self-management support, disease management; medication management and education; electronic prescribing
  - Communication and coordination: Communication with patients; coordination of care and communication among staff/team; coordination, communication, and getting appointments with other providers and specialists; referral and use of community resources; provider is informed / up-to-date about care from other providers; electronic access and sharing of information with other providers, hospitals, EDs, specialists
  - Follow-up: Tracking of care, test and lab results, referrals; alerts for providers when patients are hospitalized or for needed services; post-discharge follow-up.

***Continuity***

Types of continuity:

- Relational continuity:
  - Enables the delivery of personalized care to patients which is cost-effective (Freeman and Hjortdahl, 1997). It may enable the GP to consider the biopsychosocial factors related to their patient, understand the patient’s context and make a multi-dimensional diagnosis (Freeman et al., 2001).
  - It is reported to be associated with increased satisfaction in both the patient and the staff, increased compliance of patients to preventive regimens and increased support of ‘wait and see’ strategy, improved problem recognition and quality of management and reduced conflicts of responsibilities for clinicians (with clear accountability) (Freeman and Hughes, 2010).
- While both relational and management continuity have been reported to improve patient and staff satisfaction, there is limited evidence to support their association with improvement in clinical indicators/parameters in diabetic patients (Freeman et al., 2007).

Limitations of continuity:

- From patient perspective, longitudinal continuity may limit their choice or increase the waiting times. From the GP’s perspective, they may have limited time outside of consultations to develop other interests, work on career progression and professional development. Similarly, relational continuity might compel the GPs to do more for their patients or the patients who know their doctor well may persuade them to do more (Freeman and Hjortdahl, 1997).
- It may also encourage collusion (e.g. sickness certification), delayed diagnosis of certain conditions and compel the patient to tolerate inappropriate and detrimental waits to see their physician (Freeman and Hughes, 2010).

Degree/level of continuity:

- Continuity at practice level is good as it enables the patient to choose their preferred physician from the practice pool, but it may also “devalue the importance of doctor-patient relationship” (Freeman et al., 2003).

Cancer:

- In cancer patients, high experienced continuity of care was associated with better quality of life and lower psychological distress (Freeman et al., 2007).

Enablers of relational continuity (Freeman and Hughes, 2010):

- Providing information to the patients about importance of relational continuity, practice’s policy on continuity of care, clinicians available in the practice and the availability of consultation slots, including face-to-face, telephone or email contact.
- Clinicians and reception staff knowing who the preferred GP for the patient is.
- Ensuring sufficient duration for each consultation to enable relationship building.
- Providing access arrangements to the patient that enable them to choose who to consult, method of consultation and speed of access.
- Providing additional help to vulnerable patients who may experience access difficulties.
- Successful continuity arrangements may require identification of population for whom the provider is responsible (e.g. via a population registry) (Starfield, 1998).

Enablers for management continuity (Freeman and Hughes, 2010):

- Full use of practice information systems and electronic communication.
- Timely availability of relevant clinical information.
- Ensuring personal and regular contact between professionals in different care settings.
- Established protocols and care pathways including routines for handovers and exchange of information.
- Pro-active follow-up of patients after a significant life event.

Outcomes (Baker at al., 2020)

- There is an association between lower mortality rates and higher continuity in the context of primary care.
- This association is variable and not always present, possibly because the presumed benefits of continuity differ among different patient groups.
- The mechanisms by which continuity may achieve lower mortality rates are unknown and further research is needed to elucidate when and how continuity helps people.
